# Dental dam clamp adaptation method on carved gypsum cast

**Published:** 2014

**Authors:** NCE Cazacu

**Affiliations:** * CNC Dental Art, Bucharest, Romania; “Carol Davila” University of Medicine and Pharmacy”, Faculty of Dentistry, Bucharest, Romania

**Keywords:** Dental dam, clamp adaptation, carved gypsum cast, safe isolation, clamp elements

## Abstract

**Rationale.** Dental Dam is the safest and most efficient isolation technique in endodontics and restorative dentistry, but it also used in esthetics, orthodontics, prosthetics, pedodontics and periodontology (for teeth immobilization).

While in most cases the standard clamps are efficient, in some clinical situations clamp adaptation is mandatory in order to assure a tight contact on the tooth.

**Objective.** The purpose of this study is to list the elements of the clamp, which should be modified in order to assure a secure constriction of the clamp on the anchor tooth, by using the carved gypsum cast method.

**Methods and Results.** 100 patients were examined, diagnosed and treated for various diagnoses like simple decay, gangrene, chronic apical periodontitis, and endodontic retreatments. The clamps used in this study were produced by Hu-Friedy, Hygenic, KKD, SDI, Hager & Werken.

In 10 cases, the anchor tooth did not provide enough stability to the standard clamp – as provided by the producer. Therefore, we have done some adjustments to some of the elements of the clamp: the arch, the wings, the plateau, the active area, and the contact points. In 6 cases, major clamp adaptations on carved gypsum cast were imperative.

**Discussion.** The classic clamps cannot provide a grip to be enough in all the clinical cases due to the huge variety and position and implantation of the anchor teeth. Therefore, in such situations, the clamps should be adapted in order to provide stability and assure the safe isolation during the treatment.

The modified clamps will be useful in similar cases, so they must be kept.

## Introduction

Dental Dam is considered the safest and most efficient isolation technique in endodontics and restorative dentistry, but it is also used in esthetics, orthodontics, prosthetics, pedodontics and periodontology (for teeth immobilization).

While in most cases the standard clamps are efficient, in some clinical situations clamp adaptation is mandatory in order to assure a tight contact on the tooth.

Various authors have described clamp adaptation methods, either through clinging at normal temperature or through thermal transformation [**1**,**3**,**4**].

However, in some clinical situations, such methods do not allow a fast and efficient adaptation; therefore, the clamp adaptation method on carved gypsum cast has been imagined.

The method consists in clinging and trimming the clamp’s contact points in order to improve the clamp’s architecture, so that it offers stability and a secure tight contact on the tooth.

## Materials and method

The study included 100 cases that were performed under rubber dam isolation with the following types of treatments: adhesive methods crown restorations, endodontic treatments (chemo-mechanical root canal fillings), coronal-radicular restorations reinforced with fiberglass posts.

Casework teeth included coronary lesions Class I - V Black, teeth with massive crown destructions and root residues with good potential prosthetic restoration.

From the point of view of the pathologies, cases that require a good isolation have been selected: treatment by means of adhesive, simple pulp lesions and complicated pulp lesions with periodontal lesions.

The classic isolation systems with the following elements have been used: dental dam (thin and medium thickness, colors in shades of green, blue and gray), removable metal frame ("U" shaped - Young type) and plastic frame, metal clamps (standardized and adapted, produced by Hu-Friedy, Hygenic, KKD, SDI, Hager & Werken), rubber dam punch, rubber dam clamp forceps, tissues (with skin protection role thanks to absorbent effect), lubricants (for lips and soft parts), vacuum cleaners (normal and for endodontics use), ligatures (dental floss) and additional sealants (classical and light cure) - in some cases [**4**,**5**].

If the tooth’s cervical contour has not provided a good stability for the chosen standard clamp, it was modified by using the method on carved gypsum cast.

The method consists of the following steps:

- examining tooth and control cervical gingival sulcus width and depth by visual inspection and by using a probe; 

- local periodontal treatment: scaling and sanitation; 

- preparing impression by inserting an impregnated eviction cord (astringent solution) with an appropriate size to the cervical sulcus;

- impression taken with silicone materials (putty and fluid consistency) on a small segment (tooth and adjacent teeth area);

- the study model casting; 

- using a pencil, the cervical contour is drawn on the gypsum cast, tangential to the gingival margin;

- with an appropriate instrument, the gingival margin is engraved on the gypsum cast. This reveals the gingival fornix to ensure an optimal access and visibility over the cervical soft side hidden to the clinical examination of the gingival sulcus; 

- by using a compass it has to be measured on the cervical area - buccal and oral – the distance between the projection of the clamp contact tips; the values found in millimeters should be noted; 

- choosing the appropriate clamp, with optimal characteristics for the application on the tooth to be restored, assessing the following elements: bow, jaws, plateau, fingers, wings, active contour;

- adapting the active contour and the contact tips of the clamp by grinding at medium speed, with a stone mounted. To adapt the active contour level, high-speed rotations and extrahard burs can be used;

- checking the adapted clamp by applying with the relaxed bow (the clamp’s bow was previously lightly clung) on the gypsum cast, in order to avoid cervical scratching. Thus, the measurement (performed before using the compass) of the distance between the active contact points of the same jaw will be checked;

- if there were minor changes of the bow tension, the bow will be slightly activated by clinging and then the procedure will be continued by the clinical stage of isolation with the rubber dam.

If the clamp suffers major adaptations, the thermal treatment is mandatory [**4**].

## Results

**Fig. 1:**
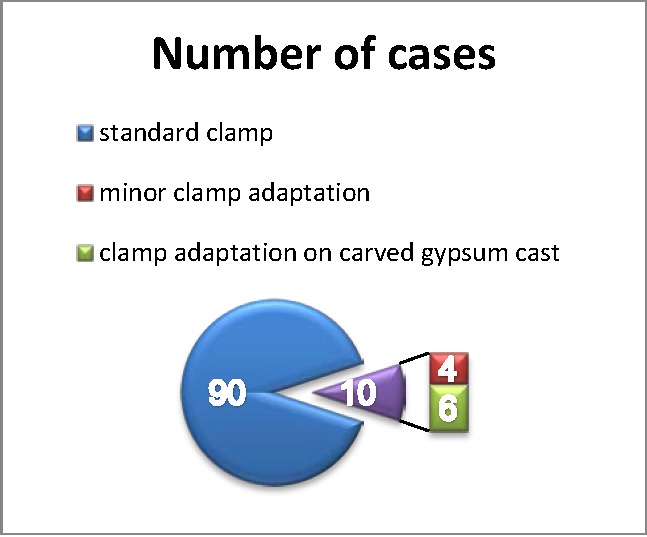
In 10% of cases the clamps requested adaptations: 4% minor adaptation and 6% major clamp adaptation on carved gypsum cast.

Of the 100 cases selected for this study, in 10 of them (10%), standard clamp applied on the tooth could not ensure a secure and stable tight contact. Of these, in 4 cases the clamp requested a minor adjustment by clinging and grinding. However, major adaptations of the clamp, the reshaping of contact points, active contour and spurs were required in 6 cases. These changes were made by using the method of clamp adaptation on the carved gypsum cast(**Fig. 1**).

## Discussions

Special situations, which may require clamp adaptation in order to assure a tight contact on the tooth, are the following:

- abnormally shaped tooth;

- inclination of the axis of teeth implantation;

- undeveloped teeth; 

- insufficient erupted teeth;

- teeth with gingival retractions; 

- teeth with irregular cervical contours, post coronary elongation; 

- teeth were prepared in order to offer support for dentures;

- teeth with massive coronal destructions that affected the cervical area and required periodontal treatment.

**Minor adaptation by clinging at room temperature**

**Case 1 standard clamp**

3.7 mesial simple decay; final restoration; clamp SDI 201 with lateral spur. The buccal spur orientation has been modified.

**Fig. 2:**
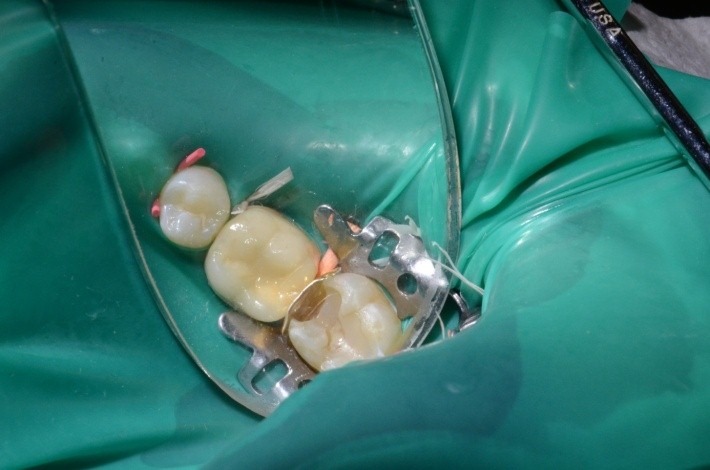
Clamp, dam, cervical ligatures on teeth 3.6 and 3.7, matrix, wedge; occlusal view; class II atypical cavity; group isolation

**Minor adaptation by clinging at room temperature and grinding**

**Case 2 standard clamp modified**

1.2 coronal fracture; root canal treatment; clamp 212 with 2 bows (butterfly); adaptations: re-shaping the contact points and jaws’ orientations.

**Fig. 3:**
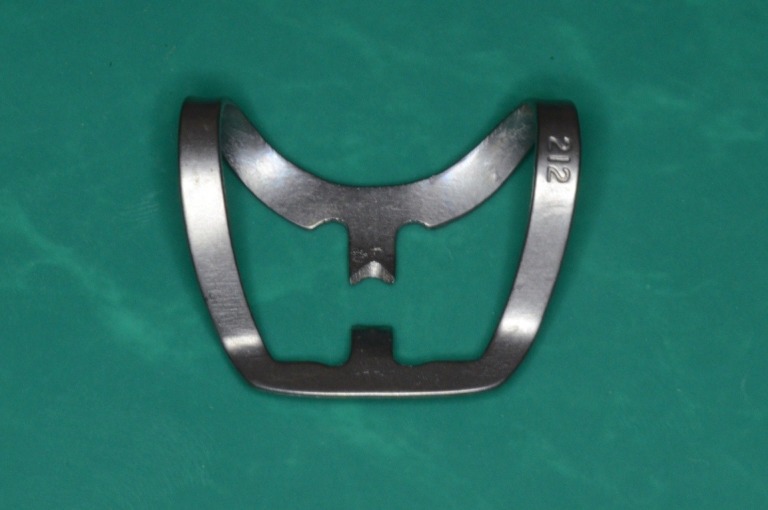
Clamp 212 standard

**Fig. 4:**
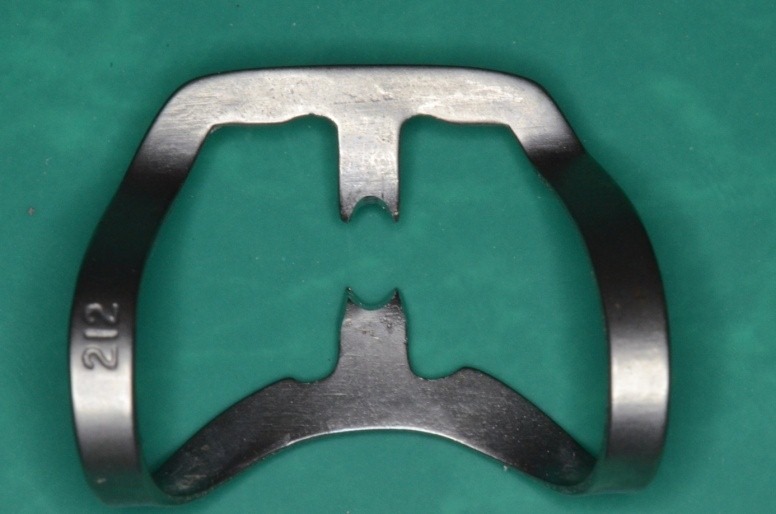
Clamp 212 modified

**Fig. 5:**
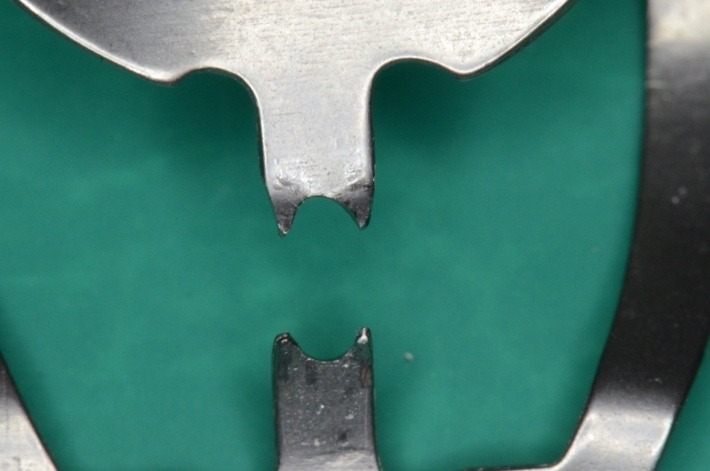
Detail: re-shaping the active perimeter; the contact points are close to one another

**Fig. 6:**
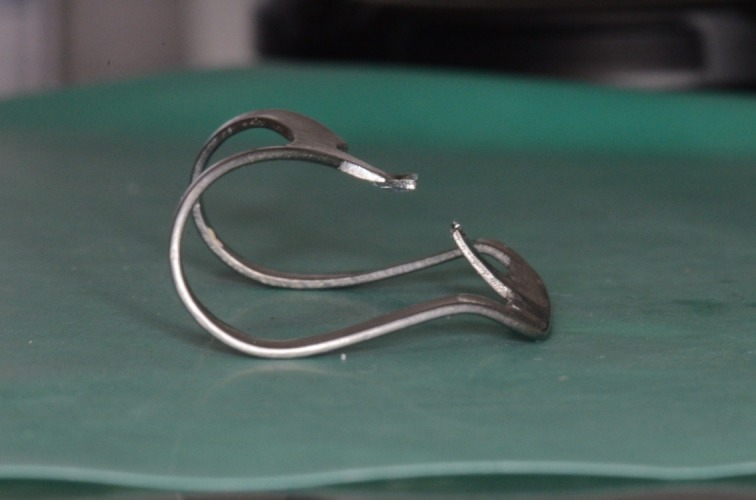
Clamp 212 modified by changing the jaws’ orientation (method described in the specific literature [**1**,**2**]).

**Fig. 7:**
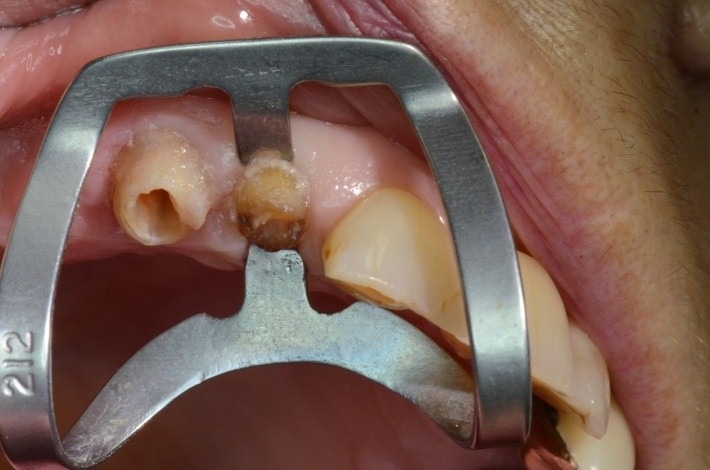
1.2 coronal fracture; root canal treatment; the clamp applied

**Fig. 8:**
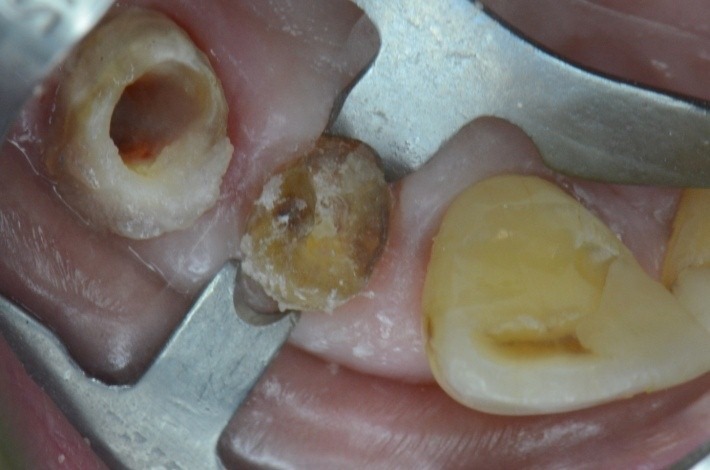
The clamp applied; detail, in the mirror: the contact area section. What is important to be noticed is the position of the active contact points on the cervical contour

**Fig. 9:**
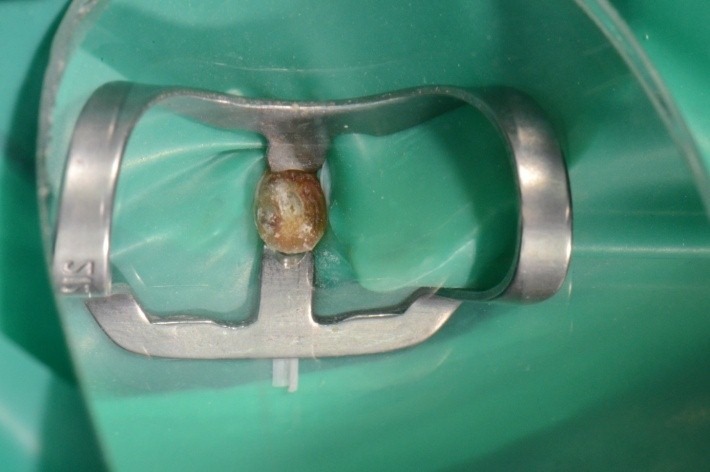
Clamp, dam, cervical ligatures

**Minor adaptation by clinging at room temperature and grinding**

**Case 3 Standard clamp modified**

4.6 simple buccal decay

Clamp SDI 210 butterfly

**Fig. 10:**
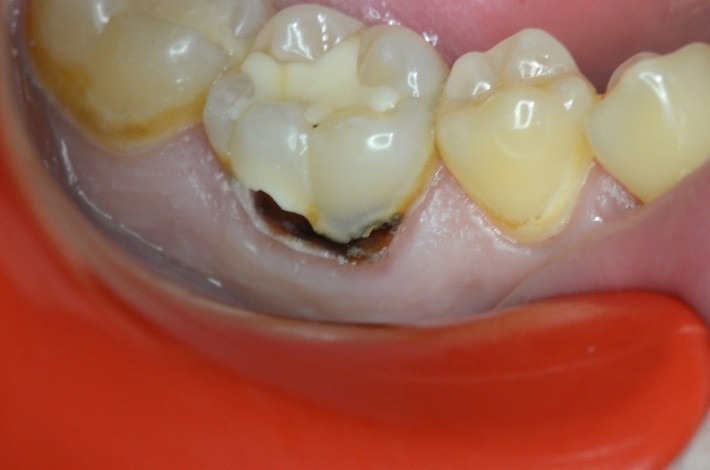
4.6 simple buccal decay

**Fig. 11:**
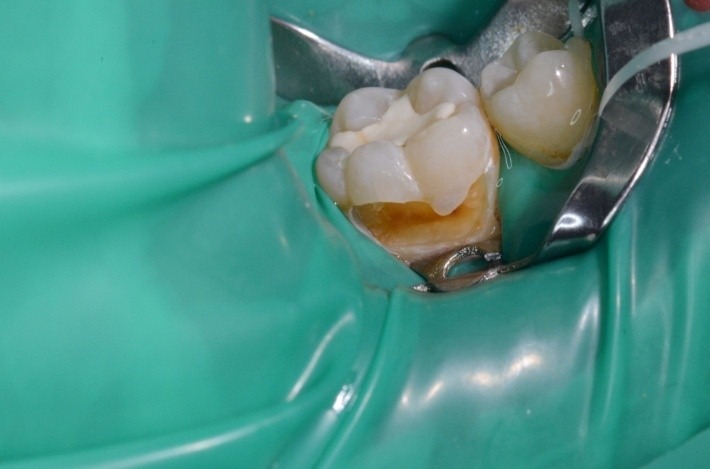
4.6 simple buccal decay; clamp and dam applied; the buccal clamp contour pushes the gingiva, offering visibility to the hard dental tissue

**Fig. 12:**
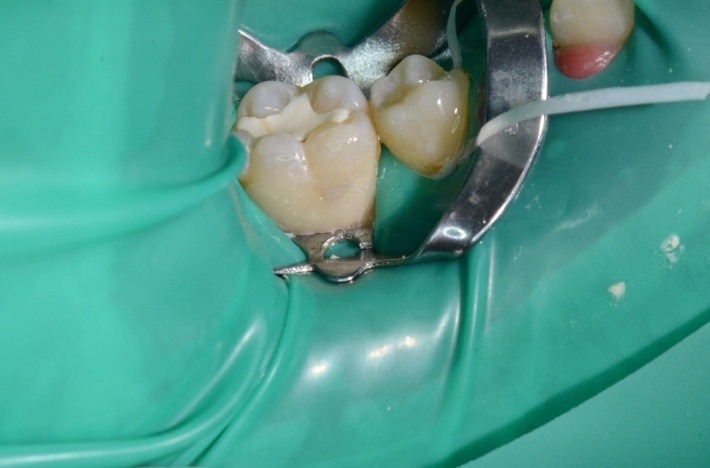
4.6 unpolished filling

**Minor adaptation by clinging at high temperature and grinding**

**Case 4 standard clamp adapted**

2.6 massive coronal destruction; crown restoration reinforced with fiberglass posts

**Fig. 13:**
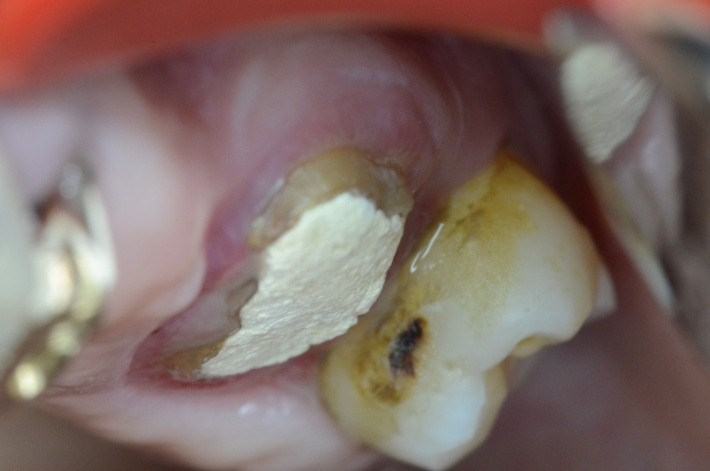
2.6 massive coronal destruction; gingivectomy performed with electro cauterization – image after the healing

**Fig. 14:**
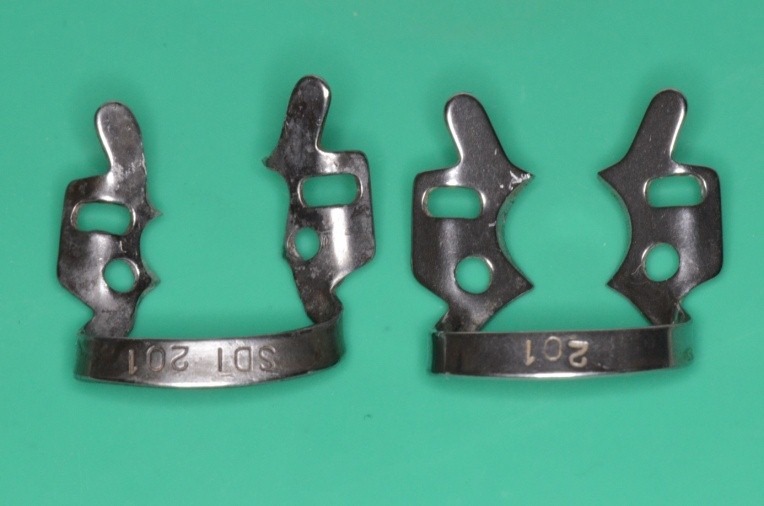
Clamp 201: left - standard; right – modified at the level of the following elements: bow, plateau, active perimeter, contact points and jaws translation

**Fig. 15:**
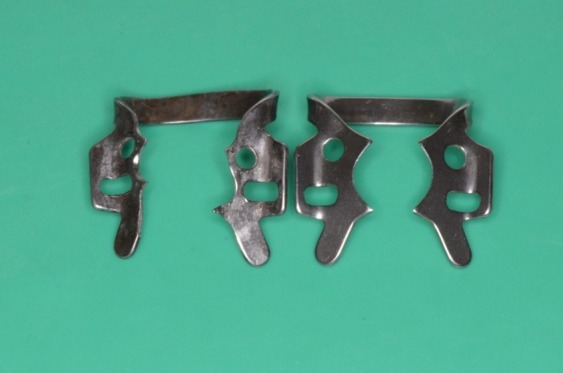
Clamp 201 – mucosal view

**Fig. 16:**
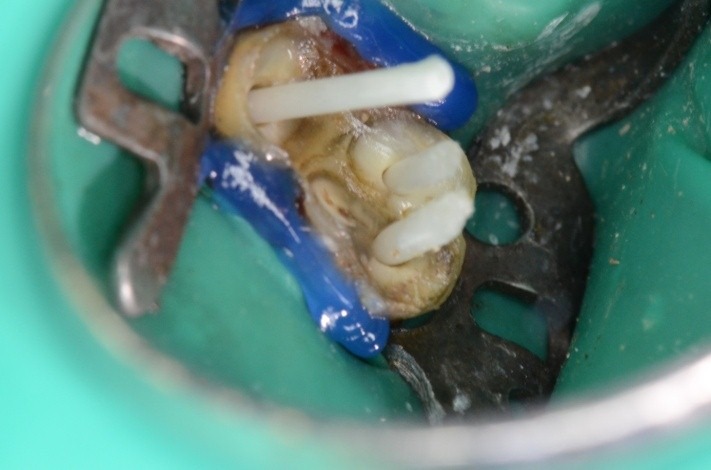
Clamp, dam, ligature; supplementary sealer; fiberglass posts checkup and adaptation

**Fig. 17:**
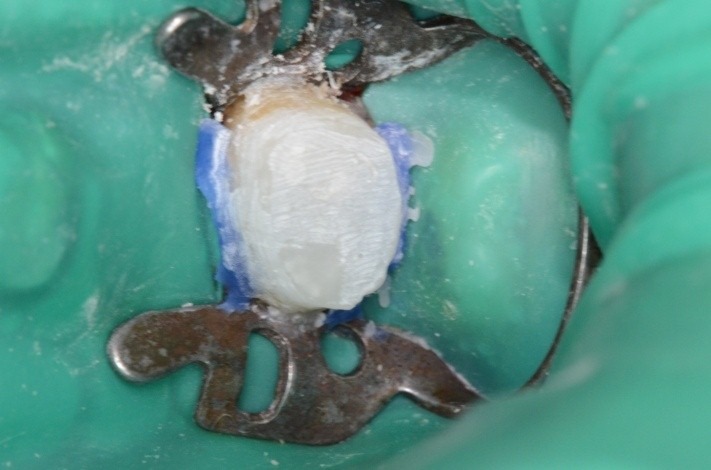
Reinforced restoration with fiberglass posts; clamp 201 modified; supplementary sealer: Block Out Resin Ultradent

**Fig. 18:**
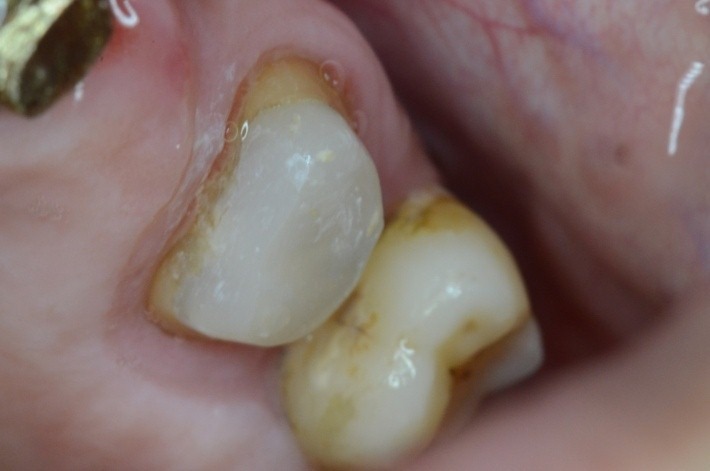
2.6 restoration ready to be prepared for the prosthetic reconstruction

**Minor adaptation by clinging and grinding**

**Case 5 standard clamp adapted**

1.4 massive coronal destruction; gingivectomy

**Fig. 19:**
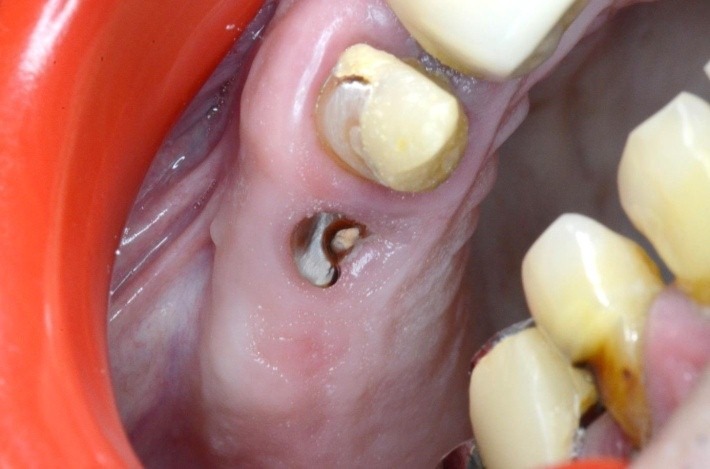
1.4 massive coronal destruction; applying a standard clamp is difficult.

**Fig. 20:**
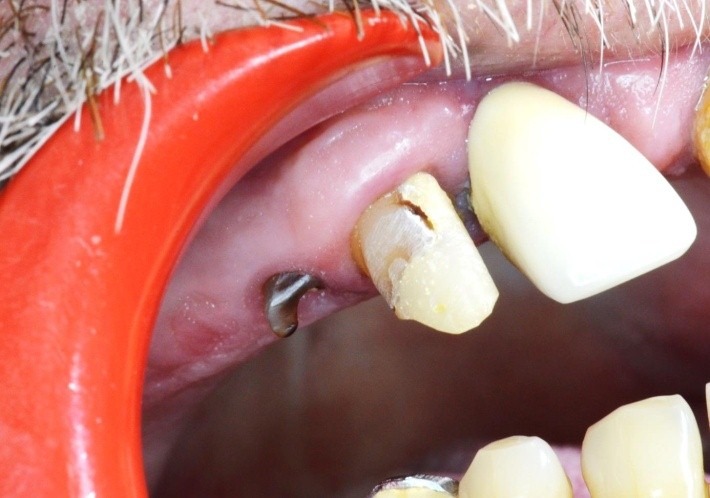
Buccal view

**Fig. 21:**
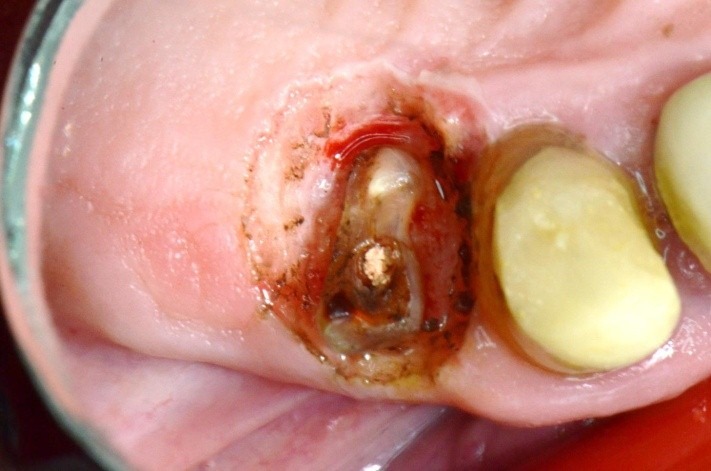
After the gingivectomy performed with electrocauterisation – occlusal view

**Fig. 22:**
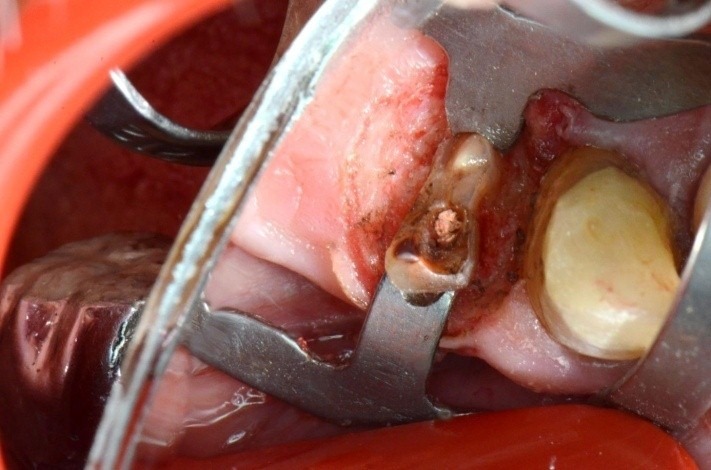
The adapted clamp has been applied

**Fig. 23:**
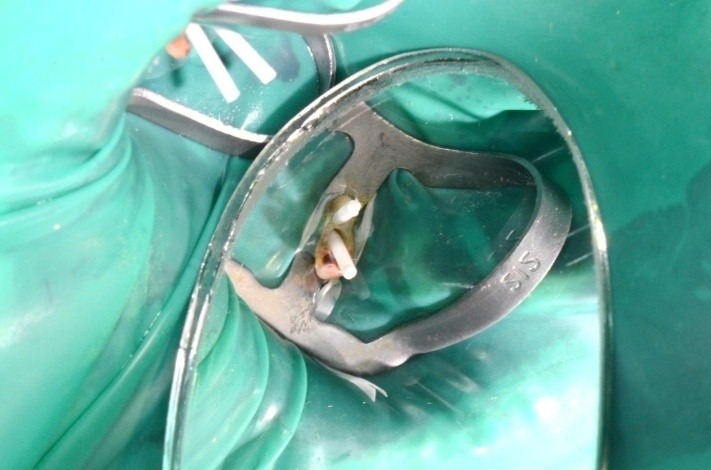
Fiberglass posts checkup

**Fig. 24:**
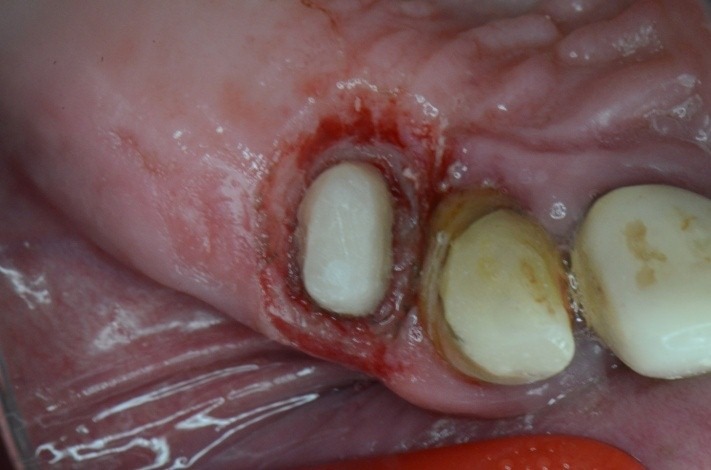
Occlusal view over the restoration after the dental dam has been removed

**Fig. 25:**
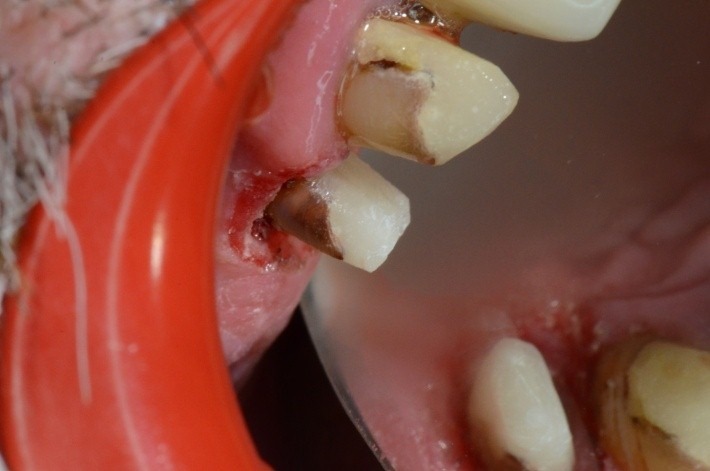
Occlusal view over the restoration after the dental dam has been removed

**Clamp adaptation method on carved gypsum cast**

**Case 6 clamp adapted on carved gypsum cast**

Elements of the clamp that have been adapted: active contour and contact points. 

1.6 grinded tooth, incomplete root canal treatment, via falsa in the floor of the pulp chamber.

**Fig. 26:**
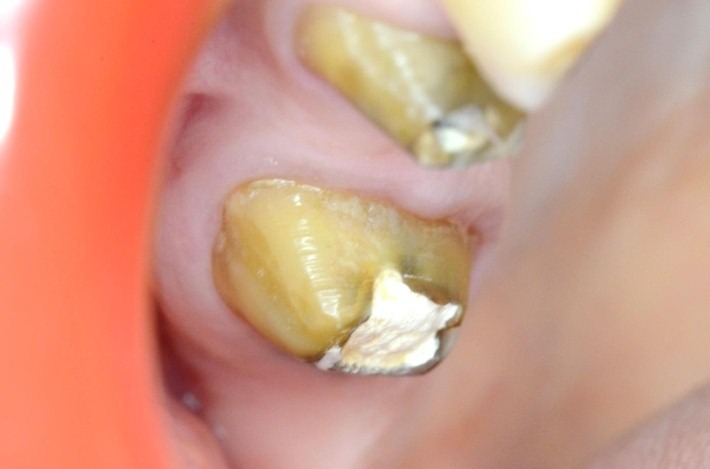
1.6 grinded tooth, incomplete root canal treatment, via falsa in the floor of the pulp chamber

**Fig. 27:**
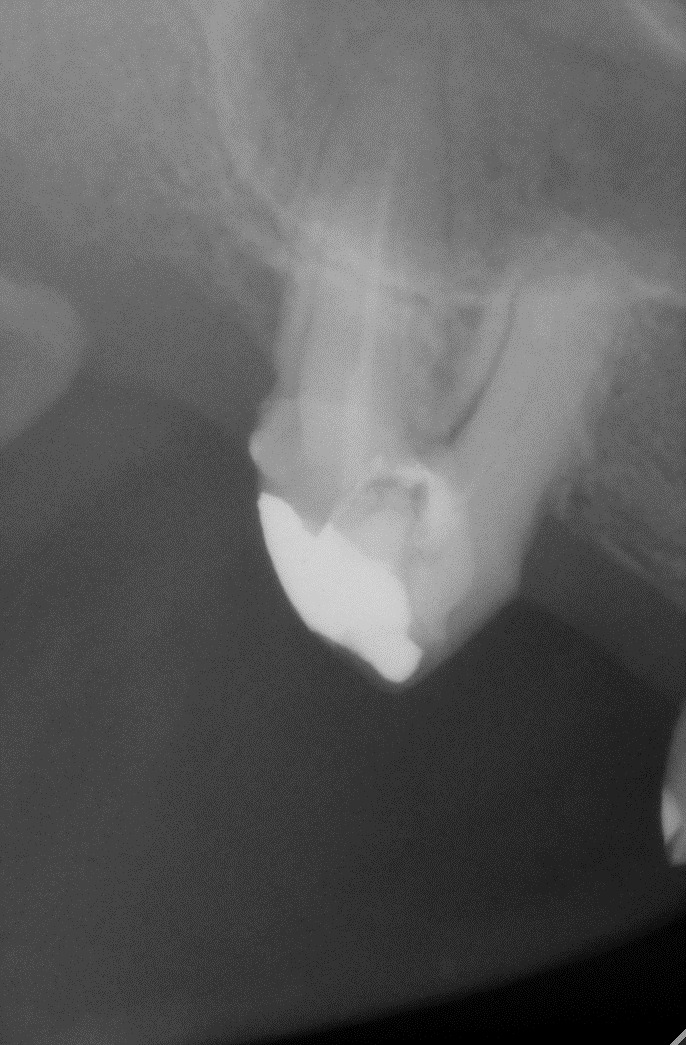
1.6 radiography realized before the treatment; incomplete root canal filling; via falsa in the floor of the pulp chamber incorrect filled; inflammation still present

**Fig. 28:**
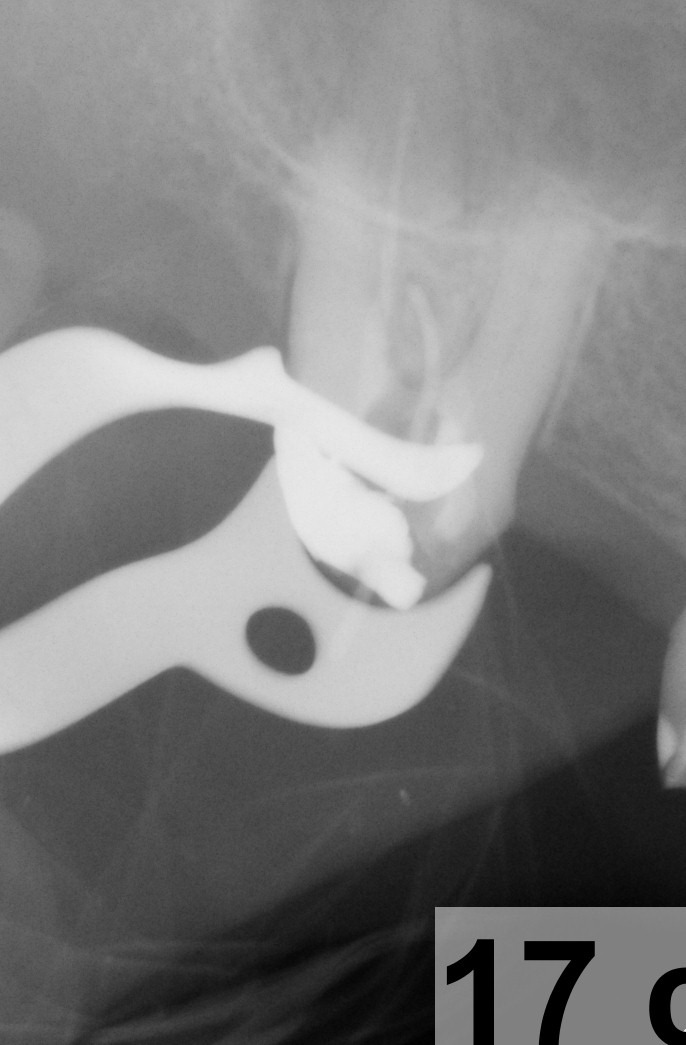
Via falsa in the floor of the pulp chamber highlighted by the gutta percha points

**Fig. 29:**
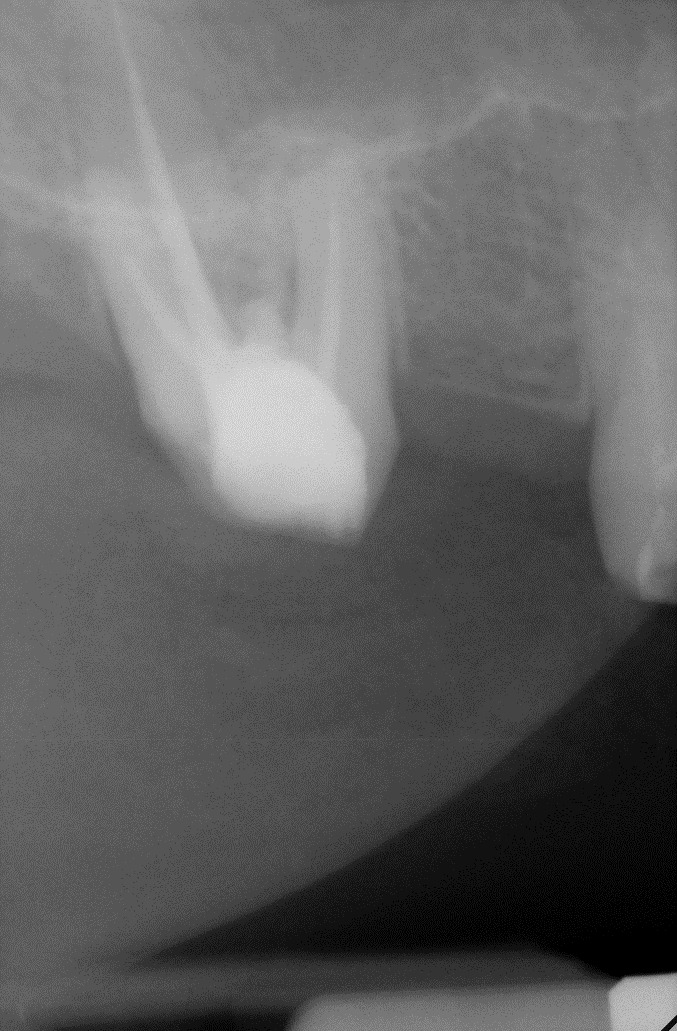
1.6 Retreatment, via falsa has been filled by using MTA; final radiography

**Fig. 30:**
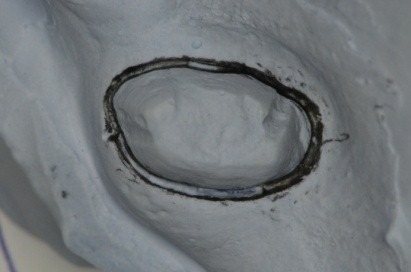
Marking the gingival contour on the gypsum cast

**Fig. 31:**
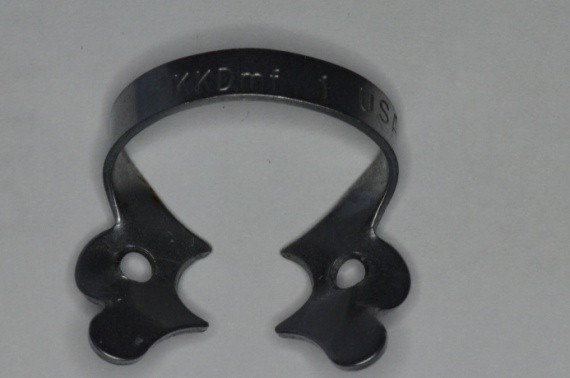
Standard clamp no. 1, KKD for upper premolars, being used unconventionally for the molar, because it has the elements which are most appropriate for the requirements of the clinical situation

**Fig. 32:**
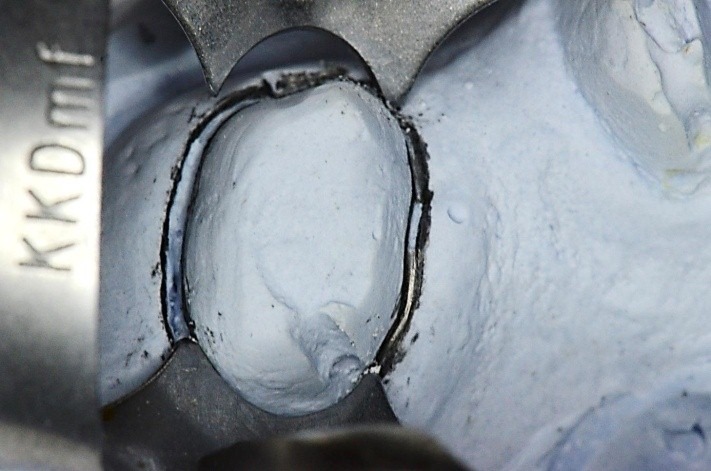
Choosing the clamp with the most appropriate contour. It is obvious the buccal-distal contact point is not in contact with the tooth on the gypsum cast. The grip is not efficient!

**Fig. 33:**
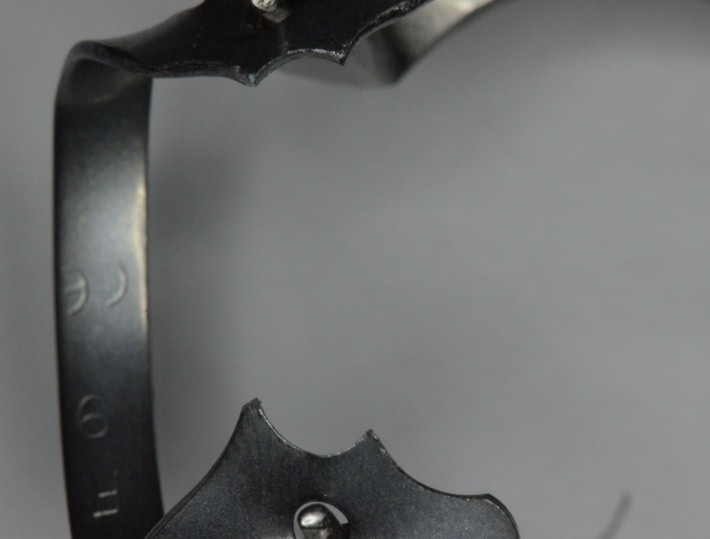
Clamp no. 1 modified: active contour and contact points

**Fig. 34:**
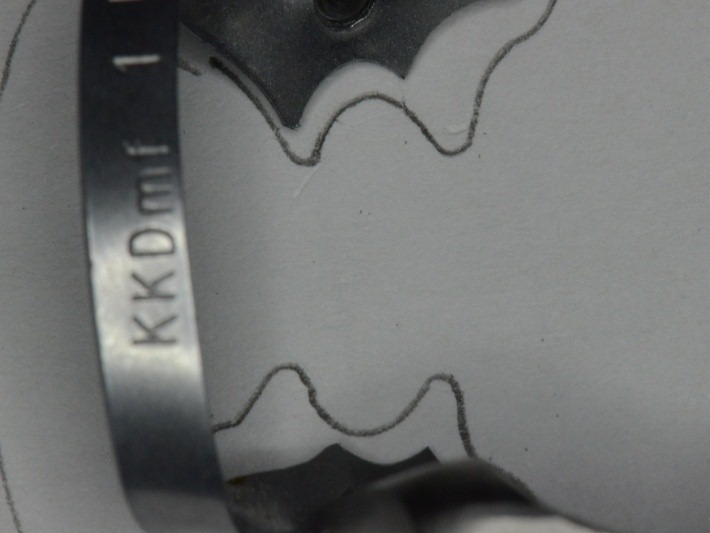
Notice the difference between the initial contour of the standard clamp (drawn on the paper) and the modified one, according to the real contact section

**Fig. 35:**
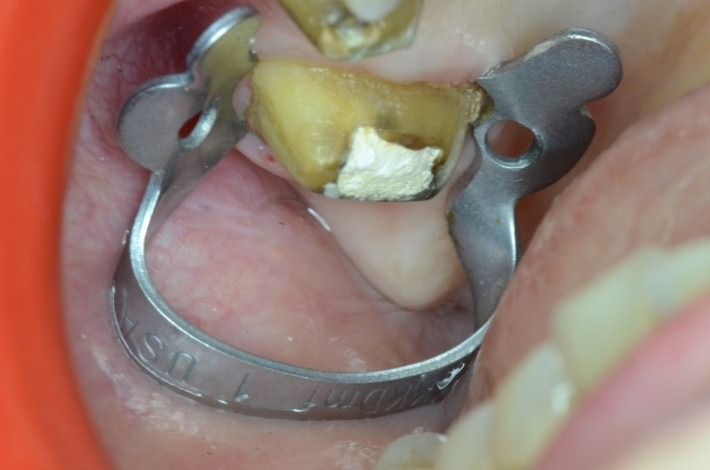
Clamp no.1 for the upper premolar adapted has been applied on the grinded 1.6. Retreatment, via falsa correctly filled

**Sources of Funding:** None

**Disclosures:** None

**Fig. 36:**
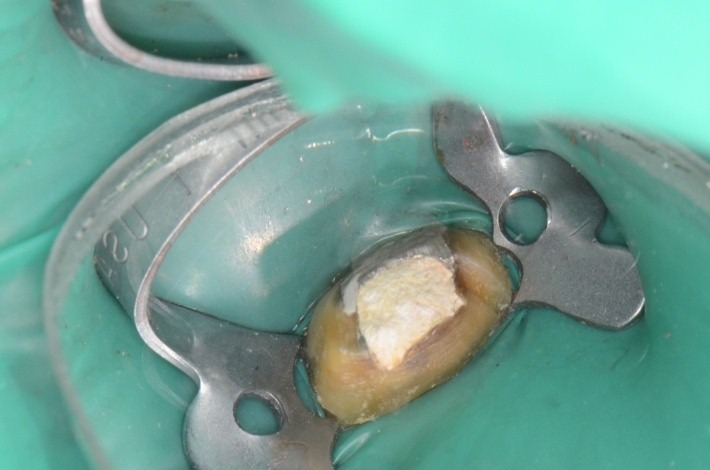
Clamp and dam – the contact points are now in the right position, so they offer good stability during treatment
